# Antiviral Effects of Heparan Sulfate Analogue‐Modified Two‐Dimensional MXene Nanocomposites on PRRSV and SARS‐CoV‐2

**DOI:** 10.1002/anbr.202200067

**Published:** 2022-08-15

**Authors:** Ting Tong, Wantao Tang, Shaobo Xiao, Jiangong Liang

**Affiliations:** ^1^ College of Science College of Resource and Environment State Key Laboratory of Agricultural Microbiology Huazhong Agricultural University Wuhan 430070 P. R. China; ^2^ College of Veterinary Medicine State Key Laboratory of Agricultural Microbiology Key Laboratory of Preventive Veterinary Medicine in Hubei Province Huazhong Agricultural University Wuhan 430070 P. R. China

**Keywords:** antiviral mechanisms, heparan sulfate analogues, PRRSV, SARS-CoV-2, two-dimensional MXene nanocomposites

## Abstract

Due to the worldwide impact of viruses such as SARS‐CoV‐2, researchers have paid extensive attention to antiviral reagents against viruses. Despite extensive research on two‐dimensional (2D) transition metal carbides (MXenes) in the field of biomaterials, their antiviral effects have received little attention. In this work, heparan sulfate analogue (sodium 3‐mercapto‐1‐propanesulfonate, MPS) modified 2D MXene nanocomposites (Ti_3_C_2_‐Au‐MPS) for prevention of viral infection are prepared and investigated using severe acute respiratory syndrome coronavirus 2 (SARS‐CoV‐2) pseudovirus and porcine reproductive and respiratory syndrome virus (PRRSV) as two model viruses. Ti_3_C_2_‐Au‐MPS nanocomposites are shown to possess antiviral properties in the different stages of PRRSV proliferation, such as direct interaction with PRRS virions and inhibiting their adsorption and penetration in the host cell. Additionally, Ti_3_C_2_‐Au‐MPS nanocomposites can strongly inhibit the infection of SARS‐CoV‐2 pseudovirus as shown by the contents of its reporter gene GFP and luciferase. These results demonstrate the potential broad‐spectrum antiviral property of Ti_3_C_2_‐Au‐MPS nanocomposites against viruses with the receptor of heparin sulfate. This work sheds light on the specific antiviral effects of MXene‐based nanocomposites against viruses and may facilitate further exploration of their antiviral applications.

## Graphical abstract

Heparan sulfate analogue (sodium 3‐mercaptopropane sulfonate, MPS) modified MXene nanocomposites (Ti_3_C_2_‐Au‐MPS) are successfully synthesized, which can strongly inhibit porcine reproductive and respiratory syndrome virus (PRRSV) and severe acute respiratory syndrome coronavirus 2 (SARS‐CoV‐2) pseudovirus. The results demonstrate the potential broad‐spectrum antiviral property of the as‐prepared Ti_3_C_2_‐Au‐MPS nanocomposites against the virus with the receptor of heparin sulfate.

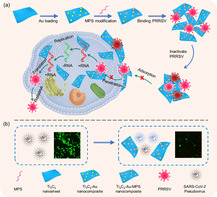

## 1 Introduction

Viruses associated with respiratory infection can invade and proliferate in the respiratory tract, causing respiratory diseases.^[^ ^1^ ^]^ The porcine reproductive and respiratory syndrome (PRRS) is a disease with the symptoms of poor growth performance, weight loss, high fever, late‐term abortion, high morbidity, and high mortality in pigs of all ages.^[^ ^2^ ^]^ PRRS virus (PRRSV), the causative agent, is a single‐stranded positive‐sense RNA virus of Arterivirus, which is very vulnerable to mutation, resulting in genetic variations within genotypes.^[^ ^3^ ^]^ Strategies such as herd closure, vaccination, and live virus exposure could not prevent the spread of PRRS disease at international, national, regional and herd levels, leading to huge economic losses in the pig industry worldwide.^[^ 4, 5, 6 ^]^ Moreover, vaccines are usually directed against a specific virus species and unable to provide adequate protection due to viral mutation accumulation and therapeutic escape.^[^ ^7^ ^]^ The COVID‐19 pandemic triggered by severe acute respiratory syndrome coronavirus 2 (SARS‐CoV‐2) has become a worldwide health disaster.^[^ ^8^ ^]^ SARS‐CoV‐2 has been shown to be the most widespread virus in modern human history, creating an immense burden on health care systems and causing enormous economic losses globally, thus highlighting the potential harm of highly infectious respiratory viruses to public health due to their infectious properties.^[^ 8, 9 ^]^ The application of vaccines has failed to prevent the continuous outbreak of this disease, leading to an upsurge in antiviral research.^[^ ^10^ ^]^

Heparin sulfate, a universal component of cell surface and extracellular matrix, was shown to be highly negatively charged and partially sulfated in its carbohydrate portions.^[^ ^11^ ^]^ Cell surface heparan sulfate was reported to be used by many viruses and parasites to infect target cells as a receptor.^[^ 12, 13, 14, 15 ^]^ The interaction of SARS‐CoV‐2 spike protein with angiotensin converting enzyme 2 (ACE2) and heparan sulfate was found to be achieved through its receptor binding domain, and docking analysis revealed adjacent binding sites between heparan sulfate and ACE2, enabling ACE2 and heparin to bind the spike protein in vitro independently, leading to the formation of the ternary complex with heparin used as scaffold.^[^ ^16^ ^]^ Moreover, heparan sulfate on the cell surface may promote the binding of the spike protein to ACE2, facilitating the virus entry into cells.^[^ ^17^ ^]^ Similarly, heparin sulfate has also been identified as an important PRRSV receptor due to its involvement in PRRSV attachment, internalization or uncoating.^[^ ^18^ ^]^ Receptors have become the targets for designing new anti‐viral agents because their sequences are more conserved than the genetic variations of viruses, and heparin has been reported as an effective target against PRRSV^[^ 13, 18, 19 ^]^ and SARS‐CoV‐2.^[^ 20, 21, 22 ^]^

Since the discovery of graphene, 2D materials have attracted increasing attention due to their superior physical, chemical and mechanical properties.^[^ ^23^ ^]^ A growing number of functionalized 2D nanomaterials have been used in antiviral research on account of their unique sharp edges, abundant surface charge, and large surface area.^[^ 24, 25, 26, 27, 28 ^]^ In 2011, transition metal carbides (MXenes) were first synthesized as a new family of 2D nanomaterials.^[^ ^29^ ^]^ Since then, the unique properties of MXene have been studied by related reserachers, and MXene and MXene‐composites have been used for in vitro and in vivo application after surface modification.^[^ 30, 31, 32, 33 ^]^ In general, MXene and MXene‐nanocomposites were modified by polyethylene glycol (PEG), polyvinylpyrrolidone (PVP) or lipid to improve its biocompatibility and stability.^[^ 34, 35, 36 ^]^ As the earliest discovered MXene material, titanium carbide (Ti_3_C_2_) nanosheets have gained tremendous traction due to their excellent physicochemical properties.^[^ 37, 38, 39 ^]^ Owing to the abundant elements of titanium and carbon in Ti_3_C_2_ nanosheets, researchers continuously evaluated the biosafety of titanium carbide in the organic body to expand its biological applications.^[^ 40, 41, 42 ^]^ Titanium carbide nanosheets can be surface‐modified to increase both biological stability and histocompatibility and reduce cytotoxicity, suggesting their potential in in vivo applications.^[^ ^43^ ^]^ Despite the applications of Ti_3_C_2_‐based nanocomposites in antitumor,^[^ ^32^ ^]^ anti‐bacteria,^[^ ^44^ ^]^ biosensing,^[^ ^45^ ^]^ virus detection^[^ ^46^ ^]^ and other fields,^[^ 47, 48, 49 ^]^ little attention has been given to the antiviral effect of Ti_3_C_2_ nanosheets.

In this study, the MAX phase Ti_3_AlC_2_ was etched and intercalated by hydrofluoric acid and tetrapropylammonium hydroxide, followed by obtaining Ti_3_C_2_ nanosheets and using the in situ reduction method to grow gold nanoparticles on the surface of Ti_3_C_2_ nanosheets. Then, 2D MXene nanocomposites (Ti_3_C_2_‐Au‐MPS) were successfully prepared by further modification of heparan sulfate analogue (sodium 3‐mercaptopropane sulfonate, MPS). The synthesized Ti_3_C_2_‐Au‐MPS nanocomposites can directly interact with PRRSV particles and inhibit viral adsorption and penetration in host cells. Similarly, Ti_3_C_2_‐Au‐MPS can strongly inhibit the infection of SARS‐CoV‐2 pseudovirus as indicated by the contents of the reporter gene GFP and luciferase. These results show the special antiviral properties of MXene‐based multifunctional nanostructures against viruses with heparin sulfate as receptor, which will trigger more explorations for the antiviral applications of MXenes.

## 2 Results and Discussion

### 2.1 Characterization of Ti_3_C_2_‐Au‐MPS Nanocomposites

Ti_3_C_2_‐Au‐MPS nanocomposites were synthesized through step‐by‐step functionalization. Firstly, the Al layer was removed by using hydrofluoric acid to etch the MAX phase Ti_3_AlC_2_, followed by adding intercalation agent tetrapropylammonium hydroxide (TPAOH) organic alkali to obtain Ti_3_C_2_ nanosheets,^[^ ^34^ ^]^ but the unmodified Ti_3_C_2_ nanosheets exhibited poor stability in physiological solutions. Taking advantages of the abundant hydroxyl groups, we used the in situ reduction method to introduce gold particles onto the surface of Ti_3_C_2_ nanosheets.^[^ ^46^ ^]^ Subsequently, the obtained Ti_3_C_2_‐Au nanocomposites were modified by MPS via the gold‐thiol bond, further enhancing the water solubility and stability of Ti_3_C_2_‐Au nanocomposites.

**Figure 1:**
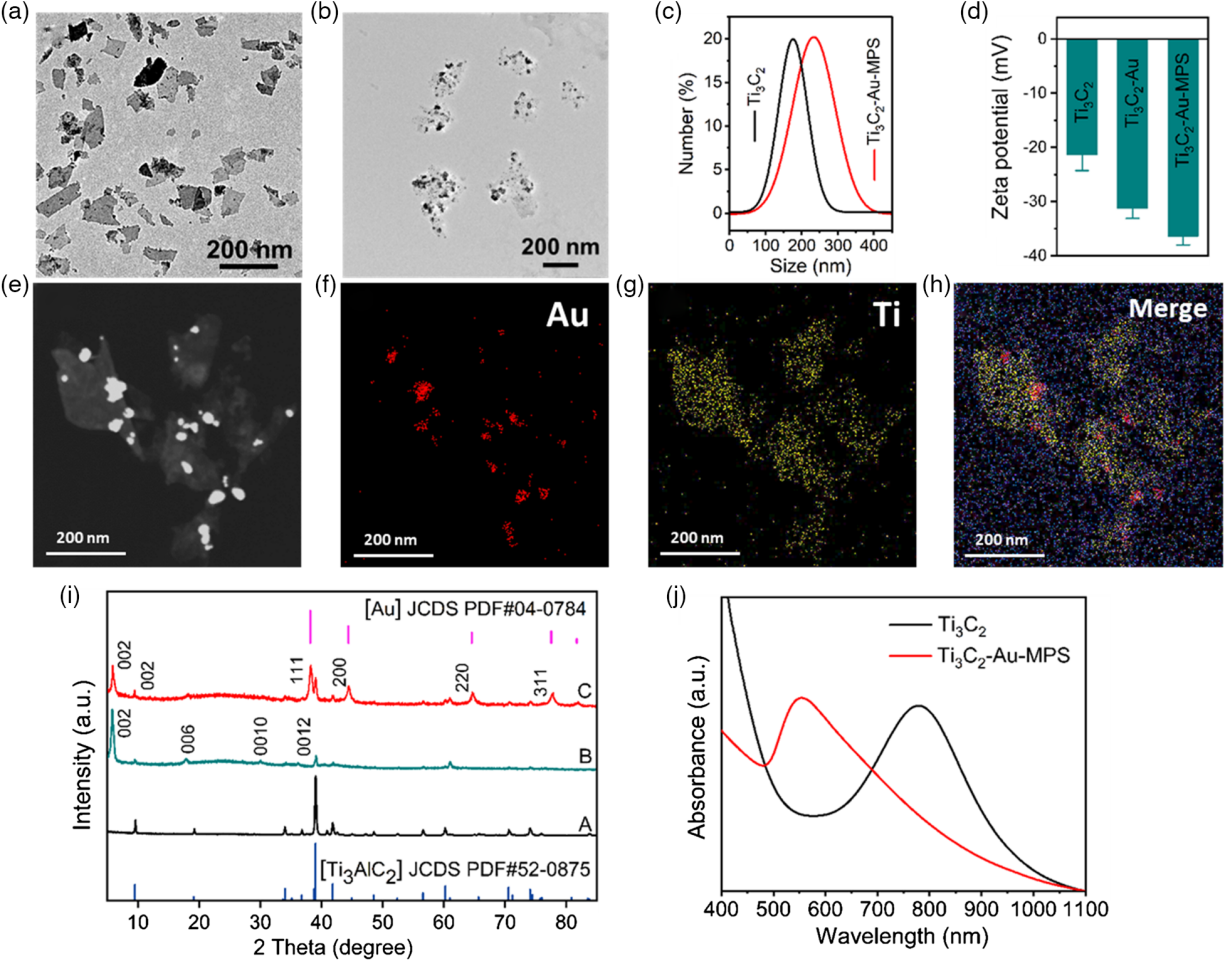
Morphology and structure characterization of Ti_3_C_2_ nanosheets, Ti_3_C_2_‐Au, and Ti_3_C_2_‐Au‐MPS nanocomposites. a) TEM image of Ti_3_C_2_ nanosheets. b) TEM image of Ti_3_C_2_‐Au‐MPS nanocomposites. c) DLS analysis of Ti_3_C_2_ and Ti_3_C_2_‐Au‐MPS nanocomposites. d) Zeta potential of Ti_3_C_2_, Ti_3_C_2_‐Au, and Ti_3_C_2_‐Au‐MPS nanocomposites. e–h) Elemental mapping of Ti_3_C_2_‐Au nanocomposite. i) XRD patterns of the samples before and after reaction in aqueous TPAOH: A, XRD pattern of synthetic raw material Ti_3_AlC_2_; B, XRD pattern of Ti_3_C_2_ nanosheets; C, XRD pattern of Ti_3_C_2_‐Au nanocomposites. j) UV‐Vis absorption spectra of Ti_3_C_2_ nanosheets and Ti_3_C_2_‐Au‐MPS nanocomposites.

The morphological characteristics of Ti_3_C_2_ nanosheets were analyzed by dynamic light scattering (DLS) and transmission electron microscopy (TEM). In **Figure** **1**a, the Ti_3_C_2_ nanosheets were seen to present a relatively uniform sheet shape. In the TEM images (Figure 1b), Au particles were seen to be distributed on the surface of the Ti_3_C_2_‐Au‐MPS nanocomposites. DLS analysis revealed the average hydrated particle sizes of Ti_3_C_2_ nanosheets and Ti_3_C_2_‐Au‐MPS nanocomposites were 176 and 211 nm, respectively, suggesting an increase in the average size after modification with Au and MPS (Figure 1c). The surface of Ti_3_C_2_ nanosheets was rich in hydroxyl groups and had a negative zeta potential, which retained negative potential after functionalization with Au particles and MPS, with a zeta potential of ≈36 mV for Ti_3_C_2_‐Au‐MPS nanocomposites (Figure 1d). Elemental mapping of Ti_3_C_2_‐Au nanocomposites revealed the uniform distribution of Ti elements in the matrix and the growth of Au particles on the surface of the nanosheets (Figure 1e–h).

The successful synthesis of  Ti_3_C_2_ nanosheets and Ti_3_C_2_‐Au nanocomposites was also confirmed by XRD. The XRD patterns of Ti_3_AlC_2_ before (curve A) and after HF stripping (curve B) are shown in Figure 1i. An obvious strong diffraction peak of (002) could be observed, and Ti_3_AlC_2_ showed a shift in the (002) peak, indicating that the bulk was stripped. In the XRD pattern, Ti_3_C_2_‐Au nanocomposites exhibited several diffraction peaks, corresponding to diffraction from the (111), (200), (220), and (311) facets of face‐centered cubic Au (curve C), indicating that the Ti_3_C_2_‐Au nanocomposites not only retained the characteristic diffraction peak of Ti_3_C_2_ nanosheets, but also presented the diffraction peaks of Au, further verifying the successful synthesis of Ti_3_C_2_‐Au nanocomposites, which agreed with the previously reported Ti_3_C_2_ complex.^[^ 34, 50 ^]^

The nanocomposites were also characterized by UV‐Vis spectroscopy. In the UV‐vis spectra (Figure 1j), the Ti_3_C_2_ nanosheets showed an absorption peak at 780 nm, and after modification with Au and MPS, the adsorption peak showed a shift from 780 to 554 nm due to surface plasmon resonance effect of Ti_3_C_2_‐Au‐MPS nanocomposites. Detailed optical images and corresponding UV‐Vis spectra of Ti_3_C_2_ nanosheets, Ti_3_C_2_‐Au nanocomposites and Ti_3_C_2_‐Au‐MPS nanocomposites are shown in Figure S1, Supporting Information. Ti_3_C_2_‐Au nanocomposites tended to aggregate in an aqueous solution, and after MPS modification, Ti_3_C_2_‐Au‐MPS nanocomposites showed very smooth absorption spectra and good dispersion in aqueous solution, indicating their prominent water solubility and the successful construction of Ti_3_C_2_‐Au‐MPS nanocomposites. The extinction coefficient of Ti_3_C_2_ was 27.0 L g^−1^ cm^−1^ (Figure S1b, Supporting Information), which was similar to the previously reported values.^[^ ^51^ ^]^

**Figure 2:**
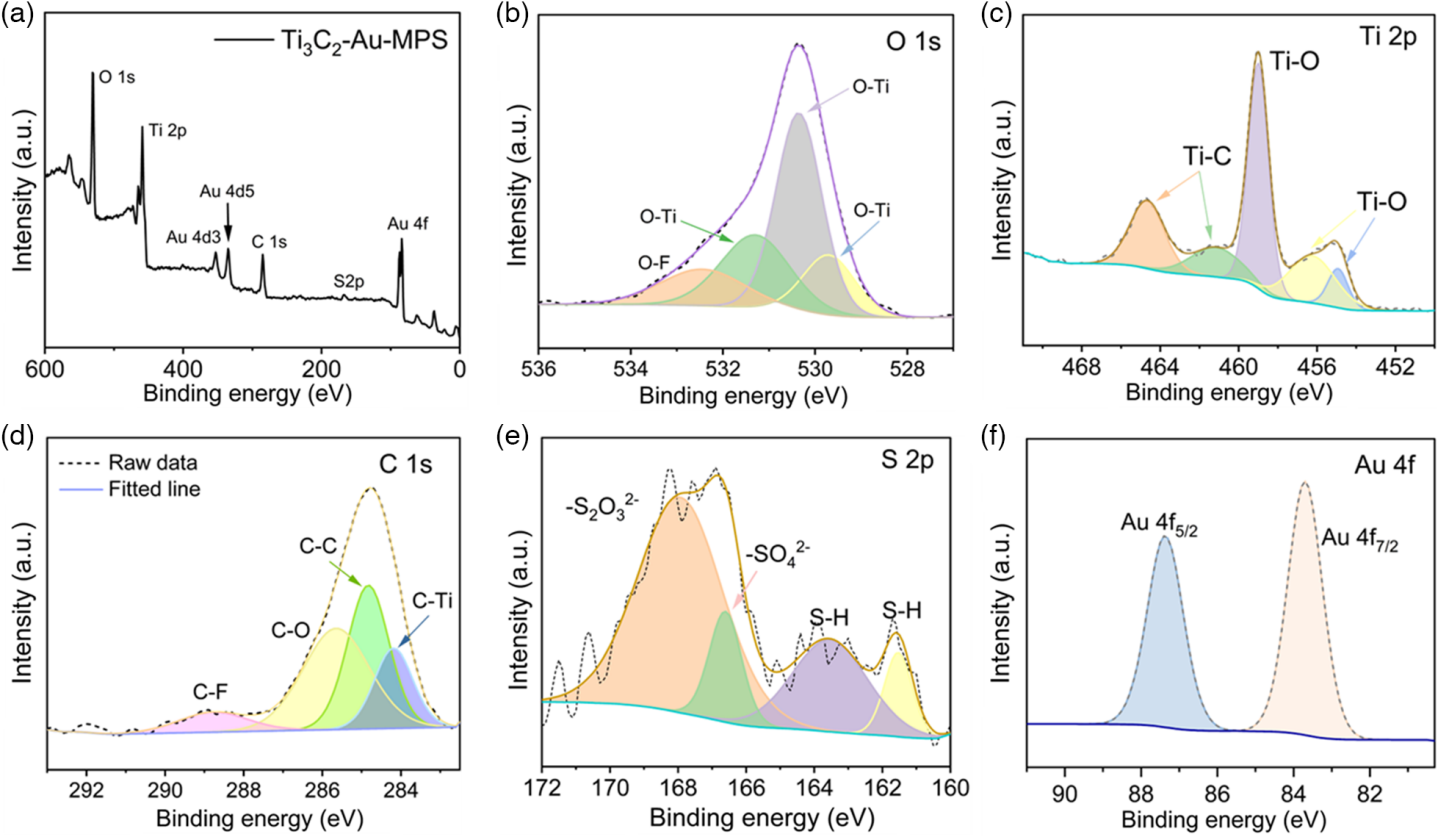
XPS spectra of Ti_3_C_2_‐Au‐MPS nanocomposites and their surface oxygen, titanium, carbon, gold, and sulfur elements. a) Full scan XPS spectrum. b–f) High‐resolution XPS spectra of O 1*s*, Ti 2*p*, C 1*s*, S 2*p* and Au 4*f* in Ti_3_C_2_‐Au‐MPS nanocomposites.

The surface chemical states of Ti_3_C_2_‐Au‐MPS nanocomposites were analyzed by X‐Ray photoelectron spectroscopy (XPS). The full survey spectra of Ti_3_C_2_ nanosheets (Figure S2a–c, Supporting Information) showed the presence of Ti, C, O and F. The presence of O and F indicated the possible surface termination of [Ti_3_C_2_(OH)_2_, Ti_3_C_2_F_2_] during etching.^[^ ^29^ ^]^ Figure S2d–f, Supporting Information, display the XPS spectra of Ti_3_C_2_‐Au nanocomposite, and the presence of Au indicates the successful growth of Au particles on the Ti_3_C_2_ nanosheet surface. **Figure** **2**a shows the XPS spectra of the pristine Ti_3_C_2_‐Au‐MPS nanocomposites, and the binding energies of Ti 2*p* and O 1*s* are consistent with the previous study.^[^ ^51^ ^]^ In Figure 2b, the O 1*s* peak at 532.5 eV represents the internal O—F bond, and the peaks at 530.3, 531.3, and 529.7 eV are assigned to the O—Ti bond, respectively. In Figure 2c, the Ti 2*p* peaks at 464.6 and 461.3 eV are attributed to the Ti—C bond, while the peaks at 459.0, 456.3, and 454.9 eV correspond to the Ti—O bond. In Figure 2d, the C 1*s* peak at 284.1 eV is attributed to the internal C—Ti bond, the peaks at 284.8 and 285.6 eV correspond to C—C and C—O bonds, and the weak peak at 288.7 eV represents the C‐F/O‐C=O group.^[^ ^52^ ^]^ After functionalization, the presence of S 2*p* in Ti_3_C_2_‐Au‐MPS nanocomposites indicated the successful modification of MPS. In Figure 2e, MPS modification was seen to cause the presence of S 2*p* peaks at 167.9, 166.5, 163.5 and 161.4 eV, corresponding to S_2_O_3_^2−^, SO_4_^2−^ (SO_3_^2−^), and ‐SH, respectively. In Figure 2f, the Au 4f peak was shown to present the same structure as the Ti_3_C_2_‐Au nanocomposites without MPS modification, indicating that MPS still retained the complete molecular structure.

These results comprehensively prove the successful synthesis, good dispersion, and excellent morphological characteristics of Ti_3_C_2_‐Au‐MPS nanocomposites.

### 2.2 Cytotoxicity and Antiviral Activity Assay of Ti_3_C_2_‐Au‐MPS Nanocomposites

**Figure 3:**
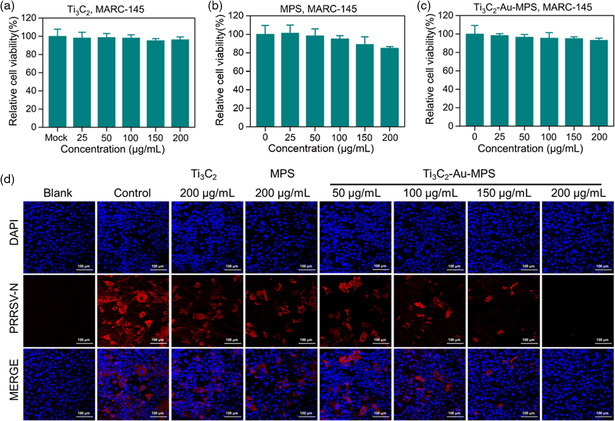
Cytotoxicity and antiviral activity of each component. a,b,c) Cytotoxicity of different concentrations of Ti_3_C_2_ nanosheets, MPS, and Ti_3_C_2_‐Au‐MPS nanocomposites (0–200 μg mL^−1^) on MARC‐145 cells by MTT assay. Error bars represent the standard deviation from three repeated experiments. All results were shown as means ± SD (*n* = 3) d) IFA images of PRRSV‐infected MARC‐145 cells treated with Ti_3_C_2_ nanosheet, MPS, and Ti_3_C_2_‐Au‐MPS nanocomposites (0–200 μg mL^−1^) for 24 hpi (MOI = 1.0). Blank group: MARC‐145 cells untreated with nanocomposites or PRRSV. Control group: MARC‐145 cells inoculated with PRRSV but untreated with nanocomposites. Scale bar = 100 μm.

Firstly, we explored the antiviral activity of Ti_3_C_2_‐Au‐MPS nanocomposites by analyzing whether Ti_3_C_2_ nanosheets or MPS can inhibit the proliferation of PRRSV. As a cell highly susceptible to PRRSV, MARC‐145 cells were selected to test the cytotoxicity of different concentrations of Ti_3_C_2_ nanosheets, MPS, and Ti_3_C_2_‐Au‐MPS nanocomposites by MTT reagent assay. As displayed in **Figure** **3**a–c, Ti_3_C_2_‐Au‐MPS nanocomposites showed no obvious cytotoxicity on MARC‐145 cells at a high concentration, and similar results were obtained in a longer time frame (Figure S3, Supporting Information). Meanwhile, the cell viability of MARC‐145 cells still exceeded 90% after incubation with Ti_3_C_2_‐Au‐MPS nanocomposites for 24 h, confirming the marginal cytotoxicity of Ti_3_C_2_‐Au‐MPS nanocomposites.

Additionally, we explored the antiviral effect of each component by indirect immunofluorescence assay (IFA). Figure 3d displays the the nucleus stained blue with 2‐(4‐amidinophenyl)‐6‐indolecarbamidine dihydrochloride (DAPI) and the viral N protein stained red with Alexa Fluor 594 dye. Ti_3_C_2_ nanosheets, small molecule MPS, and Ti_3_C_2_‐Au‐MPS nanocomposites were shown to have a certain antiviral effect at 0–200 μg mL^−1^ within 24 h post infection (*hpi*) at multiplicity of infection (MOI) of 1.0. The inhibitory effect was relatively weak for Ti_3_C_2_ nanosheets, suggesting their limited antiviral activity without modification. Meanwhile, MPS also reflected a faintish antivirus effect, which is similar to previous reports,^[^ 14, 53 ^]^ mainly because small molecules have an extremely small size and are difficult to enter cells.^[^ ^54^ ^]^ However, the MPS‐modified Ti_3_C_2_‐based nanocomposite (Ti_3_C_2_‐Au‐MPS) showed strong antiviral activity, demonstrating the advantages of MPS modification with Ti_3_C_2_ nanosheets. This result confirmed our hypothesis that Ti_3_C_2_‐Au‐MPS nanocomposites possess an effective antiviral activity.

### 2.3 Antiviral Activity of Ti_3_C_2_‐Au‐MPS Nanocomposites Against PRRSV Infection

**Figure 4:**
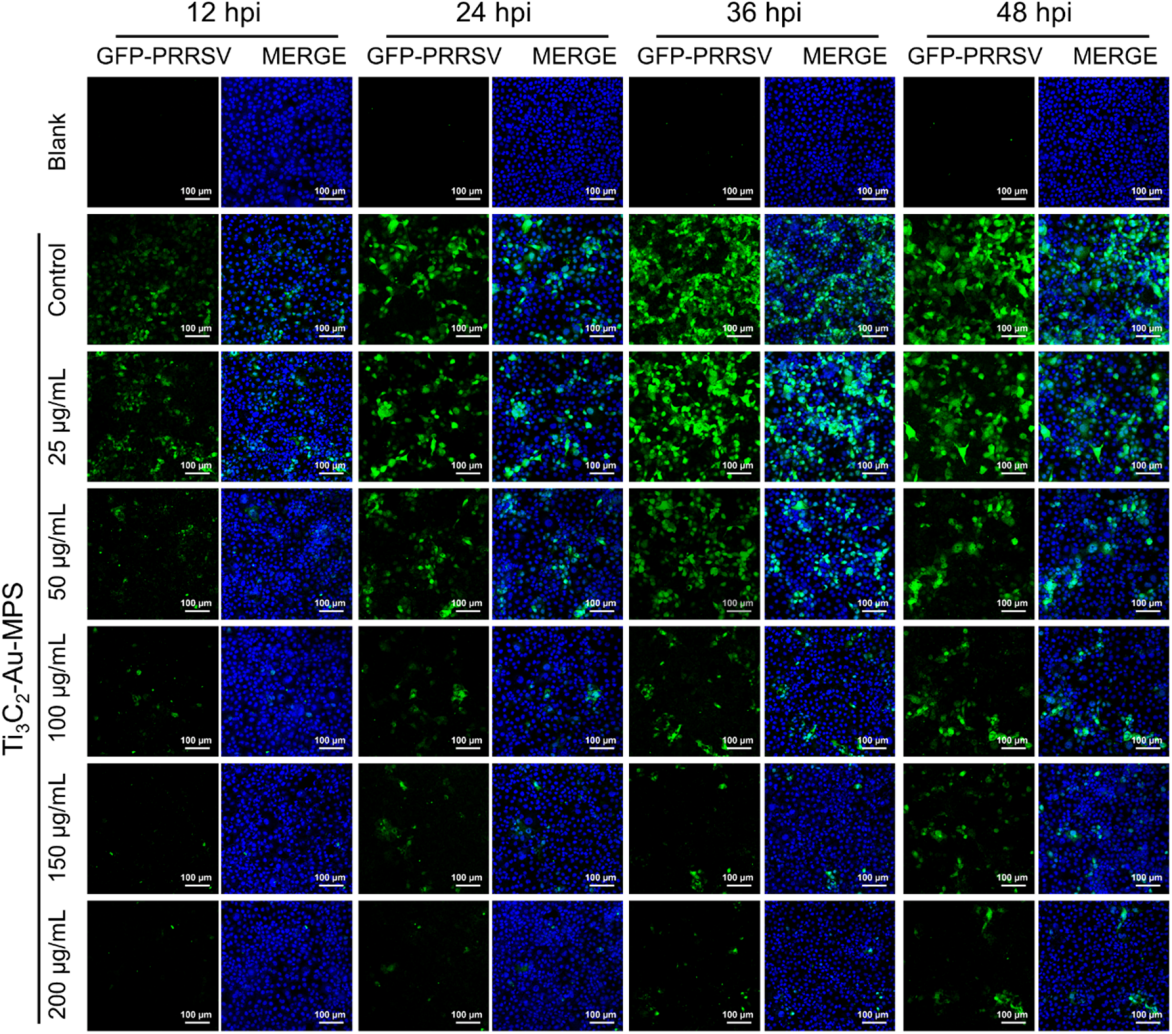
IFA images of GFP‐PRRSV (MOI = 1.0) infected MARC‐145 cells treated and untreated with Ti_3_C_2_‐Au‐MPS nanocomposites (0–200 μg mL^−1^) at 12, 24, 36, and 48 hpi, respectively, with blue for nucleus and green for GFP‐PRRSV, in a random field of view. Blank group: MARC‐145 cells untreated with nanocomposites or PRRSV. Control group: MARC‐145 cells inoculated with PRRSV but untreated with nanocomposites. Scale bar = 100 μm.

GFP‐PRRSV is a virus genetically engineered by inserting the green fluorescent protein (GFP) gene into the unstructured protein (nsp2) of the PRRSV genome, which can infect the host cell and express GFP at the same time. Therefore, the expression of GFP can be used to detect the infection amount of virus. In order to visualize the effect of Ti_3_C_2_‐Au‐MPS nanocomposites on PRRSV infection, GFP‐PRRSV was used to infect MARC‐145 cells incubated or unincubated with Ti_3_C_2_‐Au‐MPS nanocomposites, and after GFP‐PRRSV infection for 12, 24, 36 and 48 h, the amount of cell infection was analyzed with a laser confocal fluorescence microscope. In **Figure** **4**, the control group showed an increase in the content of green fluorescent protein with the extension of virus infection time, and Ti_3_C_2_‐Au‐MPS nanocomposites exhibited a more obvious time‐ and dose‐dependent inhibitory effect on GFP‐PRRSV infection, with a significant decrease of green fluorescence signals in the experimental groups, directly demonstrating that Ti_3_C_2_‐Au‐MPS nanocomposites could inhibit GFP‐PRRSV proliferation. This result is consistent with the results of the above IFA on PRRSV‐infected MARC‐145 cells (Figure 3d).

**Figure 5:**
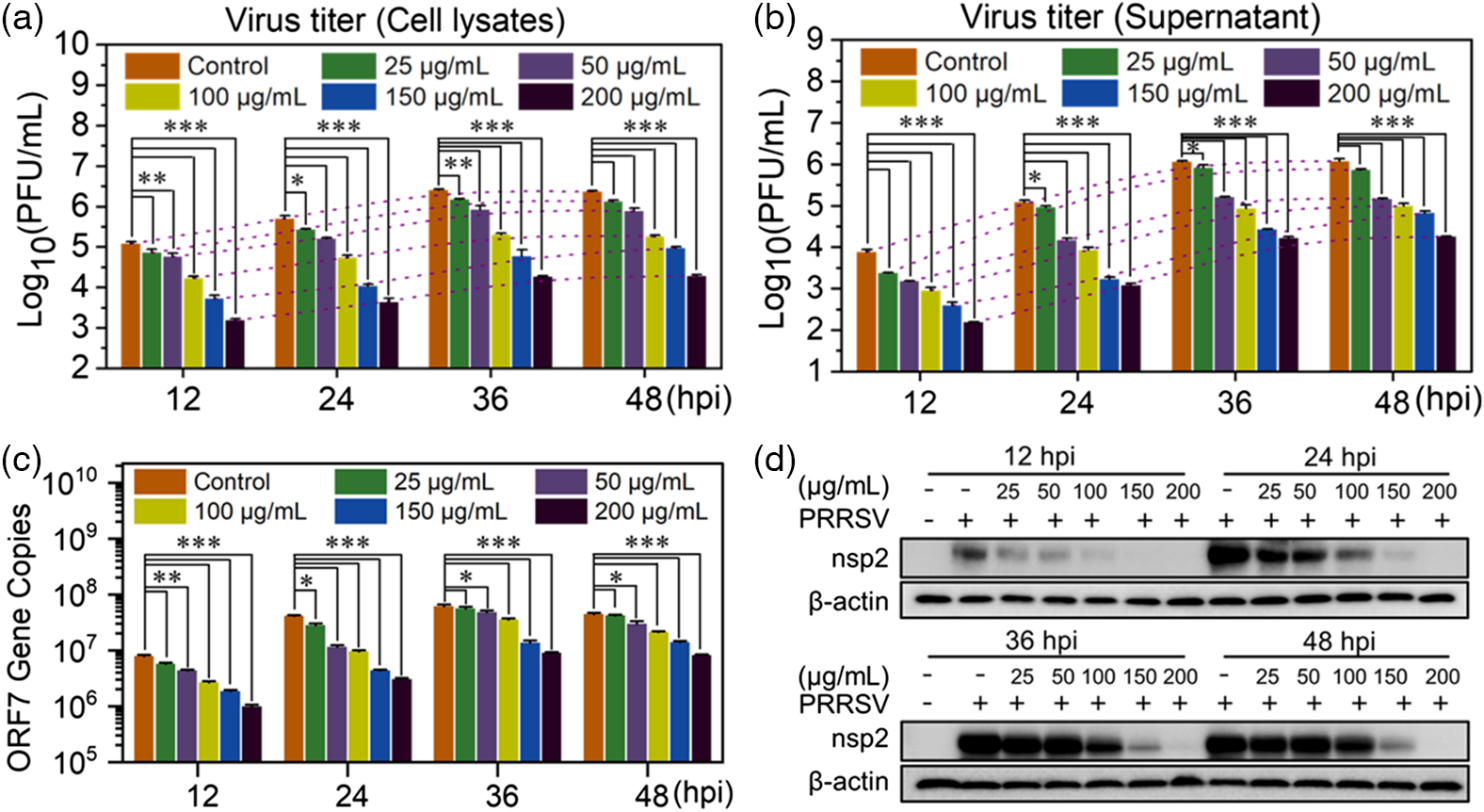
Ti_3_C_2_‐Au‐MPS nanocomposites inhibit PRRSV proliferation. Plaque reduction assay of a) intracellular and b) supernatant inhibitory effects of Ti_3_C_2_‐Au‐MPS nanocomposites on the whole replication cycle of PRRSV (MOI = 1.0). c) RT‐qPCR analysis of the content of ORF7 gene in PRRSV genome treated with different concentrations of Ti_3_C_2_‐Au‐MPS nanocomposites (0–200 μg mL^−1^). b,c) All results were shown as means ± SD (*n* = 3). *P < 0.05, **P < 0.01, ***P < 0.001. d) Western blot analysis of the expression levels of PRRSV (MOI = 1.0) nsp2 protein after treatment of Ti_3_C_2_‐Au‐MPS nanocomposites at 0–200 μg mL^−1^. Blank group (−, −): MARC‐145 cells untreated with PRRSV or nanocomposites; control group (−, +): MARC‐145 cells inoculated with PRRSV but untreated with nanocomposites.

In order to quantitatively examine the antiviral activity of Ti_3_C_2_‐Au‐MPS nanocomposites, PRRSV growth kinetics were studied by infecting MARC‐145 cells treated or untreated with Ti_3_C_2_‐Au‐MPS nanocomposites at different concentrations, followed by plaque reduction assay of the supernatant and cell lysate of the infected cells. As revealed in **Figure** **5**a,b, compared with the control group, the experimental groups treated with Ti_3_C_2_‐Au‐MPS nanocomposites showed an obvious time‐ and dose‐dependent decrease in virus titers at 25–200 μg mL^−1^, with ≈10^2^‐fold reduction, demonstrating the efficient inhibitory effect of Ti_3_C_2_‐Au‐MPS nanocomposites on PRRSV infection. The inhibition of Ti_3_C_2_‐Au‐MPS nanocomposites on PRRSV proliferation was also verified in the genome. In the single positive‐stranded RNA virus genome, there are at least 10 open reading frames (ORFs), such as ORF7, ORF6, ORF5, ORF5a, ORF4, ORF3, ORF2a, ORF2b, and ORF1a/b,^[^ ^55^ ^]^ and the cellular ORF7 gene content is related to the number of infected cells and viral infection intensity. Therefore, RT‐qPCR analysis was performed to investigate the ORF7 gene content in the PRRSV‐infected MARC‐145 cells treated with Ti_3_C_2_‐Au‐MPS nanocomposites. In Figure 5c, the genomic ORF7 content was seen to have a decrease of 1 titer in the MARC‐145 cells treated with Ti_3_C_2_‐Au‐MPS nanocomposites, further indicating that Ti_3_C_2_‐Au‐MPS nanocomposites could inhibit PRRSV proliferation by affecting genome replication.

Viral proteases were reported to have the functions of cleaving polyproteins translated from ORF1a and ORF1b into 16 nonstructural proteins (nsps): nsp1α/β, nsp2‐6, nsp7/7α, nsp8‐12, nsp2TF, and nsp2N.^[^ ^3^ ^]^ Some of the nsps could be assembled with host cell components to form replication transcription complex. This suggests that the PRRSV protein content can also reflect the effect of nanocomposites on PRRSV proliferation, so western blot analysis was used to evaluate the antiviral effect of Ti_3_C_2_‐Au‐MPS nanocomposites. PRRSV‐infected MARC‐145 cells treated with different concentrations of Ti_3_C_2_‐Au‐MPS nanocomposites were collected, followed by lysing the cells to obtain the total protein of the sample, using β‐actin as an internal control. In Figure 5d, it was shown that with the increase of infection time, the expression level of nsp2 in virus‐infected cells untreated with Ti_3_C_2_‐Au‐MPS nanocomposites showed a time‐dependent upward trend, meanwhile, the expression level of viral nsp2 in virus‐infected cells decreased in a dose‐dependent manner treated with Ti_3_C_2_‐Au‐MPS nanocomposites.

Collectively, plaque reduction assay, RT‐qPCR assay and western blot assay confirmed that Ti_3_C_2_‐Au‐MPS nanocomposites could interfere with PRRSV infectivity.

### 2.4 Ti_3_C_2_‐Au‐MPS Nanocomposites Suppress PRRSV Activity at Different Stages

**Figure 6:**
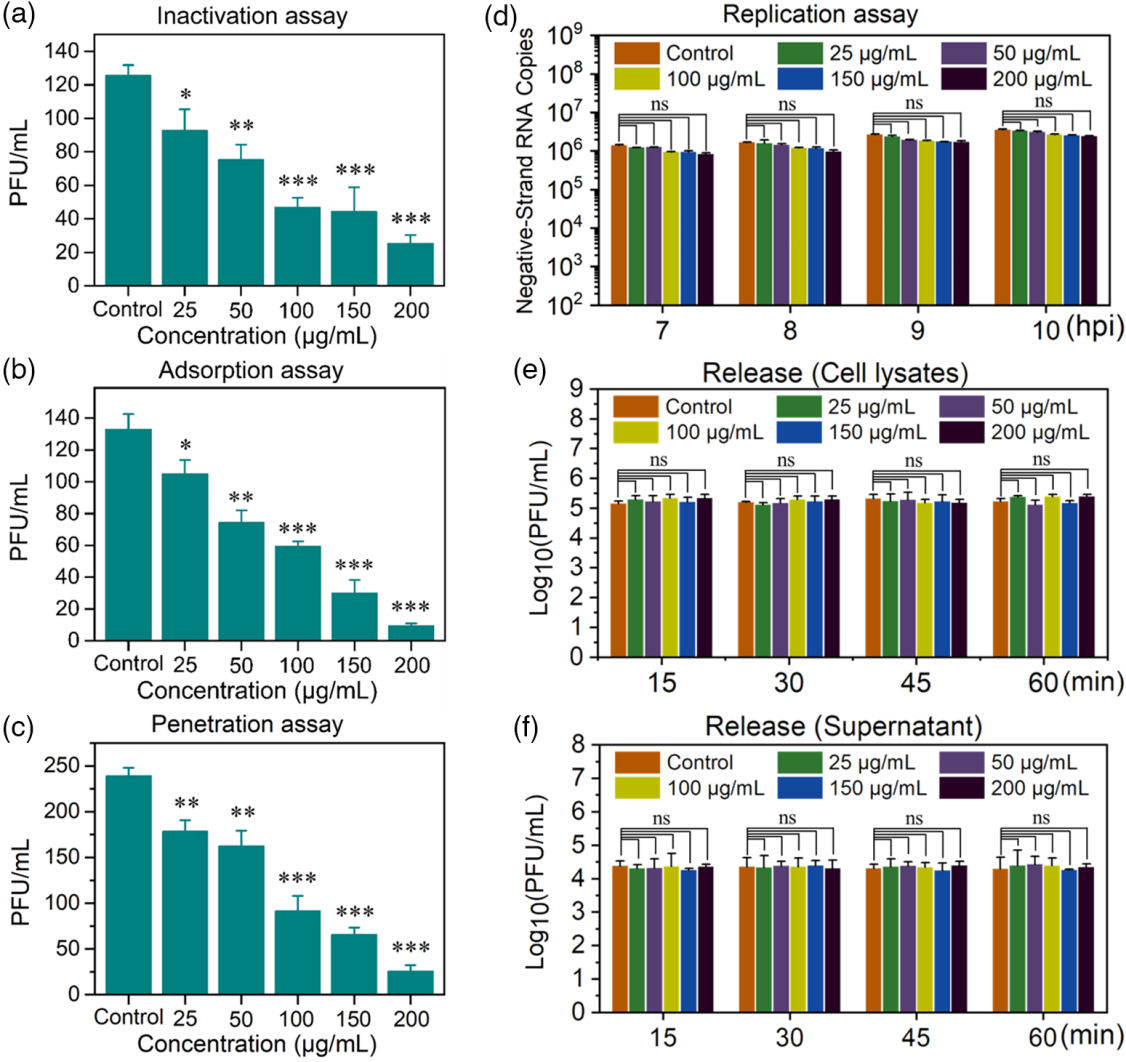
Ti_3_C_2_‐Au‐MPS nanocomposites inhibit the initial stages of PRRSV infection. a) Viral inactivation assay (MOI = 0.002). b) Adsorption assay (MOI = 0.001). c) Penetration assay (MOI = 0.005). d) Replication assay (MOI = 1.0). e,f) Release assay (MOI = 1.0). Control group: MARC‐145 cells inoculated with PRRSV, but untreated with nanocomposites. All results were shown as means ± SD (*n* = 3). *P < 0.05, **P < 0.01, ***P < 0.001.

In order to understand the details of the inactivation of PRRS virions by Ti_3_C_2_‐Au‐MPS nanocomposites, PRRS virions were extracted and purified using a sucrose density gradient centrifugation method. For simulating the interaction environment of Ti_3_C_2_‐Au‐MPS nanocomposites and PRRSV‐treated MARC‐145 cells, the purified PRRS virions were incubated at 37 °C for 1 h with Ti_3_C_2_‐Au‐MPS nanocomposites, followed by TEM analysis. In Figure S4, Supporting Information, it can be seen that PRRS virions were densely decorated with Ti_3_C_2_‐Au‐MPS nanocomposites and significantly attached to their surface, thereby deforming PRRS virions. It is reported that the tight binding of gold nanoparticles to HSV‐1 can directly affect the activity of virus particles.^[^ ^12^ ^]^ Here, the PRRSV activity was detected after the binding of Ti_3_C_2_‐Au‐MPS with virus particles, then the PRRSV infectivity was detected by plaque reduction assay, and results are shown in **Figure** **6**a. As expected, in the presence of different concentrations of Ti_3_C_2_‐Au‐MPS nanocomposites, the number of plaques decreased significantly compared to the control group untreated with Ti_3_C_2_‐Au‐MPS nanocomposites. These observations indicated that Ti_3_C_2_‐Au‐MPS nanocomposites can directly inactivate a part of PPRS virions.

Subsequently, we investigated the stages of PRRSV proliferation (adsorption, penetration, replication or release).^[^ ^50^ ^]^ Plaque reduction assay was performed and the results are shown in Figure 6b, which illustrated that Ti_3_C_2_‐Au‐MPS nanocomposites had a strong concentration‐dependent inhibitory effect on PRRSV adsorption stage. Whether Ti_3_C_2_‐Au‐MPS nanocomposites suppress viral penetration was also evaluated by plaque reduction assay. In Figure 6c, Ti_3_C_2_‐Au‐MPS nanocomposites were seen to show a strong concentration‐dependent inhibitory effect on PRRSV penetration in the cells. Whether Ti_3_C_2_‐Au‐MPS nanocomposites block PRRSV replication was investigated by RT‐qPCR analysis of PRRSV negative‐sense RNA levels. In Figure 6d, the negative‐strand PRRSV RNA expression level showed no significant difference in the cells treated or untreated with Ti_3_C_2_‐Au‐MPS nanocomposites, indicating that the Ti_3_C_2_‐Au‐MPS nanocomposites did not inhibit viral replication. Finally, whether Ti_3_C_2_‐Au‐MPS nanocomposites block the release of PRRSV was investigated. In Figure 6e,f, the cells treated or untreated with Ti_3_C_2_‐Au‐MPS nanocomposites showed no significant difference in PRRSV intracellular and supernatant titers, indicating that Ti_3_C_2_‐Au‐MPS nanocomposites did not inhibit the release of PRRSV in either supernatant or cell lysate. In general, Ti_3_C_2_‐Au‐MPS nanocomposites could inhibit the adsorption, and penetration processes of PRRSV, with an obvious reduction in the viral activities after Ti_3_C_2_‐Au‐MPS treatment. In order to show the virus plaque situation after Ti_3_C_2_‐Au‐MPS nanocomposite treatment more clearly, we took pictures of the results of an experiment (Figure S5, Supporting Information).

These findings suggested that Ti_3_C_2_‐Au‐MPS nanocomposites can interfere with the early stages of viral proliferation.

### 2.5 Antiviral Activity of Ti_3_C_2_‐Au‐MPS Nanocomposites Against SARS‐CoV‐2

**Figure 7:**
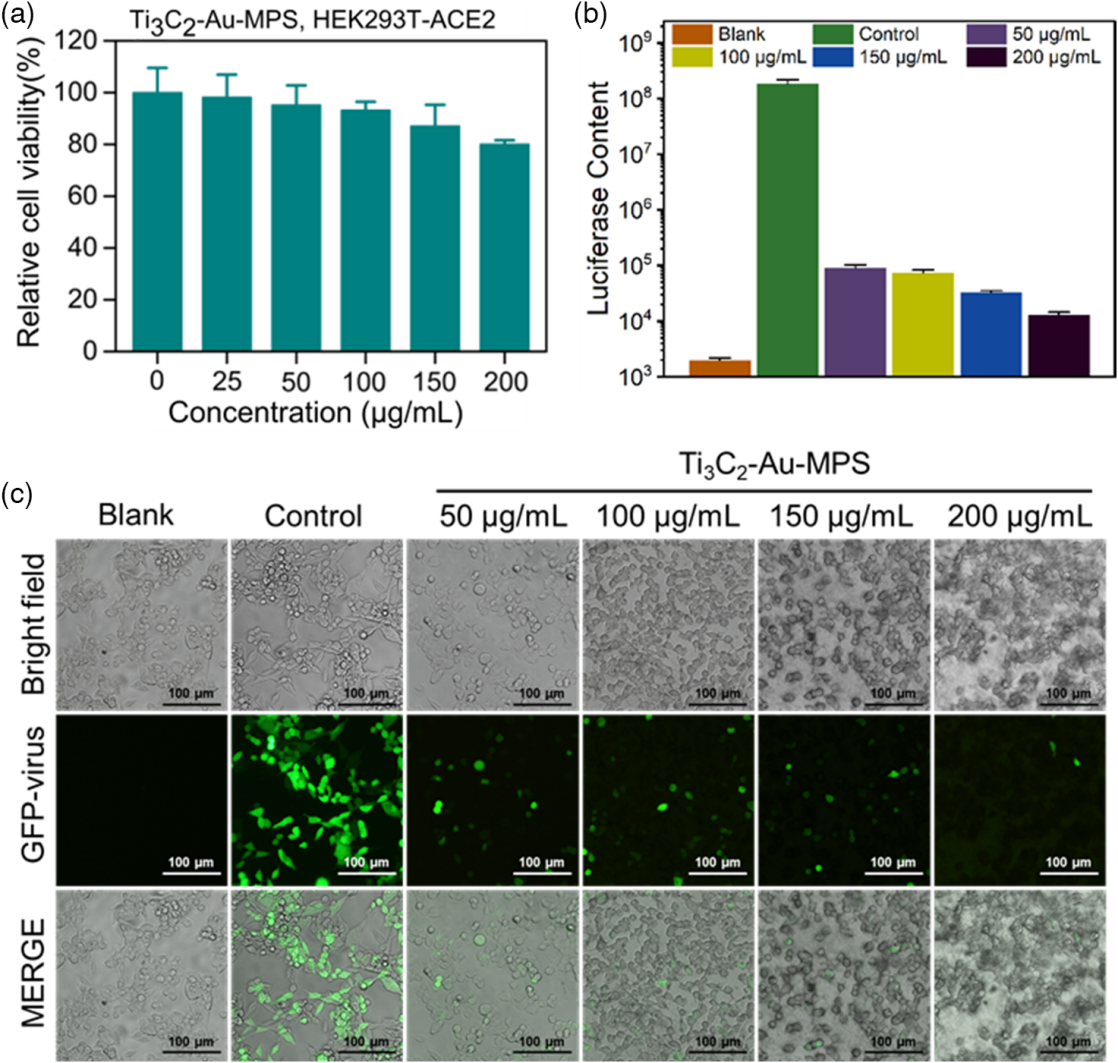
Cytotoxicity and antiviral activity of Ti_3_C_2_‐Au‐MPS nanocomposites. a) Cytotoxicity of different concentrations of Ti_3_C_2_‐Au‐MPS nanocomposites (0–200 μg mL^−1^) on HEK‐293T‐ACE2 cells by MTT assay. b) The inhibitory effects of Ti_3_C_2_‐Au‐MPS nanocomposites on SARS‐CoV‐2 pseudovirus assessed by luciferase content analysis (MOI = 5.0). (a,b) All results were shown as mean ± SD (*n* = 3). *P < 0.05, **P < 0.01, ***P < 0.001. c) Confocal images for the inhibitory effects of Ti_3_C_2_‐Au‐MPS nanocomposites on SARS‐CoV‐2 pseudovirus infection in HEK‐293T‐ACE2 cells. Blank group: HEK‐293T‐ACE2 cells untreated with nanocomposites or SARS‐CoV‐2. Control group: HEK‐293T‐ACE2 cells inoculated with SARS‐CoV‐2, but untreated with nanocomposites.

The antiviral effects of Ti_3_C_2_‐Au‐MPS nanocomposites on SARS‐CoV‐2 were interrogated by virus neutralization assay, which was reported as a sensitive strategy to assess whether neutralizers can specifically suppress SARS‐CoV‐2 infection.^[^ 16, 56, 57, 58, 59 ^]^ Using a lentivirus packaging system, the SARS‐CoV‐2 pseudovirus was created, with its spike glycoprotein in the recombinant virus envelope, and the green fluorescent protein (GFP) and the luciferase gene of the CMV promoter in the RNA genome. This facilitated the analysis of virus entry into cells by detecting GFP expression or luciferase content. After pre‐seeding into microplates overnight, HEK‐293T‐ACE2 cells were incubated with viral particles mixed with different concentrations of Ti_3_C_2_‐Au‐MPS nanocomposites (0–200 μg mL^−1^) to allow virus infection, followed by replacing the fresh medium, further culture at 37 °C for 48 h, and using the treated cells directly for fluorescence imaging. The viral infection rate was evaluated by observing GFP fluorescence intensity, then the cells were lysed and analyzed with a luciferin assay kit. **Figure** **7**a shows the relative viability of HEK‐293T‐ACE2 cells incubated with Ti_3_C_2_‐Au‐MPS nanocomposites at different concentrations, and no obvious cytotoxicity was observed for the Ti_3_C_2_‐Au‐MPS nanocomposites. In Figure 7b,c, Ti_3_C_2_‐Au‐MPS nanocomposites were seen to strongly block the SARS‐CoV‐2 pseudovirus infection at 50 μg mL^−1^ concentration. Confocal imaging results also indicated that Ti_3_C_2_‐Au‐MPS nanocomposites could protect some cells from infection, with the fluorescence intensity being obviously weaker in the infected cells treated with nanocomposites than those untreated. Overall, Ti_3_C_2_‐Au‐MPS nanocomposites provided an efficient and safe potential therapy for SARS‐CoV‐2, thus enriching the existing treatments against COVID‐19.

In recent years, the application of surface modification technology has promoted the development of antiviral nanomaterials.^[^ 12, 60, 61 ^]^ Some biomolecules can directly bind to the surface of nanomaterials for target viruses.^[^ 62, 63, 64 ^]^ In this study, combined with the excellent biocompatibility of Ti_3_C_2_ nanosheets, MPS was modified on the surface of nanosheets, enabling the functionally modified nanocomposite to have significant antiviral activity and allowing the functionalized Ti_3_C_2_ nanosheets to inhibit the proliferation of viruses with heparan sulfate as receptor or co‐receptor for the first time. In general, the nanocomposite entry inhibitor can minimize the effects of viral mutation and host toxicity without conferring immunogenicity,^[^ 65, 66, 67 ^]^ and Ti_3_C_2_‐Au‐MPS nanocomposites may also have relevant properties. Current antiviral research models mainly consider the life cycle of viruses through viral adsorption, invasion, replication, assembly, and release of new viruses,^[^ 61, 68, 69 ^]^ and Ti_3_C_2_‐Au‐MPS nanocomposites inhibit viral proliferation at every stage in the intial stage of the cycle. The first complex stage of the virus life cycle is its entry process, with virus trying to bind and enter host cell through multivalent interactions with the surface receptors and coreceptors of cell membranes. At this stage, Ti_3_C_2_‐Au‐MPS nanocomposites can function by interfering with these recognition events, thus constituting one of the most promising strategies.

With the rapid development of nanoscience in healthcare, many scientists have evaluated its side effects and toxicity in parallel. In the study by Han et al., mice were injected intravenously with 50 mg kg^−1^ of Ti_3_C_2_‐SP nanosheets for 7 days, and their major organs (heart, liver, spleen, lung, and kidney) were H&E stained, which showed that the Ti_3_C_2_‐SP nanosheets had no obvious acute toxicity and side effects, and the nanosheets were gradually excreted from the body through urine and feces with a total excretion of 18.70% and 10.35% in 48 h, respectively.^[^ ^70^ ^]^ In another systematic study of in vivo biocompatibility, 20 mg kg^−1^ of MnO_*x*_/Ti_3_C_2_‐SP was intravenously injected into healthy mice for 30 days, and their blood and major organs (heart, lung, liver, kidney and spleen) were collected for biochemical analysis. The results showed that there were insignificant changes in blood parameters, liver and kidney function indexes during the whole feeding period. Moreover, the H&E staining results showed no obvious tissue damage in the major organs after treatment with different doses of MnO_*x*_/Ti_3_C_2_‐SP.^[^ ^36^ ^]^ A similar evaluation was also performed on Ti_3_C_2_‐SP,^[^ ^33^ ^]^ which also highlighted its good biocompatibility and negligible cytotoxicity, further indicating its potential clinical translational applications. These preliminary in vivo evaluations strongly demonstrate the relatively low biotoxicity and high biocompatibility of Ti_3_C_2_‐based nanosheets, contributing to their further potential clinical translation. Despite the benignness and biosafety of MXenes composites and their approriateness for different biomedical applications shown by previously reported in vitro and in vivo studies, their clinical and translational potential needs to be further verified by detailed genotoxicity and reproductivity toxicity tests. Mehmet Altay Unal et al. analyzed the intracellular proteome changes of Vero E6 cells treated with 50 μg mL^−1^ Ti_3_C_2_T_*x*_ for 4 h. Their results indicated that Ti_3_C_2_T_*x*_ is not cytotoxic in any peripheral blood mononuclear cell population, demonstrating the high biocompatibility of Ti_3_C_2_T_*x*_. Moreover, Ti_3_C_2_T_*x*_ could reduce the release of pro‐inflammatory cytokines.^[^ ^26^ ^]^

Based on the current results of Ti_3_C_2_‐Au‐MPS at the cellular level, we are confident about the application of Ti_3_C_2_‐Au‐MPS in vivo. In the next program, we plan to use Ti_3_C_2_‐Au‐MPS for in vivo antiviral therapy, with intravenous injection as a potential delivery strategy for Ti_3_C_2_‐Au‐MPS.

## 3 Conclusions

In this study, we proposed a blocking strategy against respiratory virus (PRRSV and SARS‐CoV‐2) infection by heparan sulfate analogue‐modified MXene nanocomposites. The functional 2D nanocomposites with excellent physicochemical properties and abundant heparin analogue (MPS) demonstrated several unique advantages for antiviral research. Firstly, the Ti_3_C_2_‐Au‐MPS nanocomposites with a relatively uniform particle size and excellent biocompatibility can be synthesized in a facile method. Secondly, Ti_3_C_2_‐Au‐MPS nanocomposites can block PRRSV infection by inactivating PRRS virions in vitro and inhibiting its adsorption and invasion in host cells. Thirdly, Ti_3_C_2_‐Au‐MPS nanocomposites can strongly block SARS‐CoV‐2 infection, suggesting the broad‐spectrum antiviral activity of Ti_3_C_2_‐Au‐MPS nanocomposites against both PRRSV and SARS‐CoV‐2. Overall, we proposed a strategy for the development of promising antiviral agents against respiratory virus diseases.

## 4 Experimental Section

4.1

4.1.1

##### Preparation of Ti_3_C_2_ Nanosheets

Synthesis of Ti_3_C_2_ nanosheets followed a previous method.^[^ ^51^ ^]^ Briefly, the sample was prepared at room temperature in the whole process. First, Ti_3_AlC_2_ powers (1.0 g) were mixed with 10.0 mL of hydrofluoric acid aqueous solution (HF, AR, 40%) and stirred for 2 h, followed by centrifugal washing with ddH_2_O until neutral pH, dispersing the sample in tetra‐n‐propylammonium hydroxide (TPAOH, 25%) and stirring for 10 h. After centrifugation, the precipitate was redissolved in ddH_2_O. Finally, the mass of Ti_3_C_2_ nanosheet powder was weighed by freeze‐drying technology, and the extinction coefficient was obtained by measuring the absorption at 780 nm using a UV‐Vis absorption spectrometer.

##### Synthesis of Ti_3_C_2_‐Au‐MPS Nanocomposites

First, the purified Ti_3_C_2_ nanosheets were vigorously stirred with a mass fraction of 1% of HAuCl_4_, and when the reaction system quickly turned purple, the reaction product was defined as Ti_3_C_2_‐Au nanocomposites. The whole reaction was completed during 20 min, and the Ti_3_C_2_‐Au nanocomposites were purified by centrifugal washing three times with ddH_2_O.

Subsequently, the purified Ti_3_C_2_‐Au nanocomposites were strongly ultrasonically reacted with 15 mg mL^−1^ of MPS for 5 min, and stirred vigorously for 48 h. Finally, the products were purified by centrifugal washing three times with ddH_2_O to obtain the Ti_3_C_2_‐Au‐MPS nanocomposites.

##### Cell Viability Assay

The cytotoxicity of Ti_3_C_2_‐Au‐MPS nanocomposites on MARC‐145 cells, HEK‐293T cells, and HEK‐293T‐ACE2 cells was evaluated using the 3‐ [4,5‐ dimethylthiazol‐2‐thiazolyl] −2,5‐diphenyl tetrazolium bromide (MTT) reagent assay. The experimental method refers to previous reports.^[^ ^61^ ^]^ Briefly, after seeding in 96‐well plates, cells were incubated separately with Ti_3_C_2_‐Au‐MPS nanocomposites (0–200 μg mL^−1^) for 12, 24, 36, and 48 h. After 2 washes with PBS, the cells were cultured for 4 h at 37 °C in the fresh medium containing 20 μL MTT (5.0 mg mL^−1^), allowing MTT to reduce succinate dehydrogenase in living cell mitochondria to insoluble blue‐purple crystalline formazan and precipitate in cells. After removing the medium carefully, each well was supplemented with 150 μL dimethyl sulfoxide (DMSO), followed by shaking gently in the dark for dissolution of formazan crystals. Finally, the absorbance at 490 nm was recorded for each sample using a microplate reader for estimating relative cell survival percentage.

##### Antiviral Assay

Antiviral assay followed a previous method.^[^ ^69^ ^]^ Briefly, after seeding in 24‐well plates, MARC‐145 cells were incubated for 2 h in DMEM (2% FBS) with Ti_3_C_2_‐Au‐MPS nanocomposites (0–200 μg mL^−1^). Meanwhile, RRRSV was preincubated for 1 h at 4 °C with Ti_3_C_2_‐Au‐MPS nanocomposites (0–200 μg mL^−1^). After removing the medium containing Ti_3_C_2_‐Au‐MPS nanocomposites, each well was supplemented with the pretreated PRRSV at multiplicity of infection (MOI) of 1.0 and incubated for another 1‐2 h for infection. After supernatant removal, the cells were incubated separately with Ti_3_C_2_‐Au‐MPS nanocomposites (0–200 μg mL^−1^) for 12, 24, 36, and 48 h. Finally, cells were treated at indicated time points as required by different evaluation methods as follows:

For determination of virus content (infective titer), the supernatant was collected, followed by adding fresh culture medium, freezing and thawing the cells 3 times, lysing the cells, releasing the virus particles, and removing the cell fragments by centrifugation to collect the cell lysate. Finally, the plaque reduction method was used to determine the virus contents in the supernatant and cell lysate. For visualization of virus infection, the supernatant was removed and indirect immunofluorescence assay (IFA) was used to detect the cells. For determination of virus genome content, the supernatant was removed and the intracellular virus gene content was detected by quantitative reverse transcription‐polymerase chain reaction (RT‐qPCR). For detection of viral protein (cells were cultured in 6‐well plates to obtain sufficient protein), the supernatant was removed, and the content of viral protein in cells was collected and detected by western blot assay.

##### Multistep Inhibition Mechanism

The experimental procedure of PRRSV stepwise infection (Virucidal activity assay, Adsorption assay, Penetration assay, Replication assay, Release assay) followed the description in the literature without any modification.^[^ ^71^ ^]^

##### Isolation, Purification, Characterization, and Interaction Between Ti_3_C_2_‐Au‐MPS Nanocomposites and PRRS Virion

PRRSV was cultured in large numbers of MARC‐145 cells until obvious cytopathy could be observed under a microscope. Next, the cells were frozen and thawed 3 times, followed by centrifugation (8000 rpm, 4 °C, 30 min) to remove the cell fragments, passing the supernatant through a 0.22 μm membrane, adjusting the volume ratio of supernatant to precipitant (20% PEG‐6000, 2.5 mol L^−1^ NaCl) at 3:1, and stirring at 4 °C for 24 h. After standing overnight, the mixed solution was concentrated (12 000 rpm, 1.5 h, 4 °C), followed by supernatant removal, precipitate collection, and precipitate resuspension in 1 mL PBS. Subsequently, the virus was isolated by sucrose density gradient (30–60%) centrifugation, followed by collecting the banded virus and resuspension in 1 mL PBS.^[^ ^72^ ^]^ After incubating the purified PRRS virions with Ti_3_C_2_‐Au‐MPS nanocomposites (100 μg mL^−1^) at 37 °C for 1 h, phosphotungstic acid was used to negatively stain the PRRS virions, and transmission electron microscopic (TEM) images of PRRSV treated or untreated with Ti_3_C_2_‐Au‐MPS nanocomposites were obtained by an electron microscope.

##### Inhibitory Effects of Ti_3_C_2_‐Au‐MPS Nanocomposites on SARS‐CoV‐2 Pseudovirus Infection

Briefly, after seeding in 24‐well plates, HEK‐293T‐ACE2 cells were incubated for 2 h in DMEM medium (2% FBS) with Ti_3_C_2_‐Au‐MPS nanocomposites (0–200 μg mL^−1^). After removing the medium, each well was supplemented with the SARS‐CoV‐2 pseudovirus at MOI of 5.0, and incubated for another 8 h for infection. After supernatant removal, cells were incubated separately for 48 h with DMEM medium (2% FBS). The GFP content of SARS‐CoV‐2 pseudovirus genome was analyzed by an inverted fluorescence microscope (Nikon, Japan), and the luciferase content was detected by Dual‐Luciferase Reporter Assay System (Promega, E1910) as directed by the manufacturer.

##### Statistical Analysis

All experiments were performed independently with three repetitions and the results were shown as mean ± standard deviation (SD) (*n* = 3). All groups with significant differences to another group within the graph were indicated by asterisks (*): *p < 0.05, **p < 0.005, and ***p < 0.001. All nonsignificant results (*p* > 0.05) were denoted with “ns” in the related graph. Statistical analyses were performed in EXCEL (one‐way ANOVA, α(A): 0.05).

## Conflict of Interest

The authors declare no conflict of interest.

## Supporting information

Supplementary Material

## Data Availability

The data that support the findings of this study are available in the supplementary material of this article.
