# Supplementary material for: Antiviral Effects of Heparan Sulfate Analogue‐Modified Two‐Dimensional MXene Nanocomposites on PRRSV and SARS‐CoV‐2
Source: Adv Nanobiomed Res. 2022 Aug 15;2(10):2200067. doi: 10.1002/anbr.202200067 (PMC9538433; doi:10.1002/anbr.202200067)
Supplement: Supplementary file 1 — Supplementary Material [file ANBR-2-0-s001.pdf]

Supporting Information

Antiviral Effects of Heparan Sulfate Analogue-modified Two-dimensional MXene Nanocomposites on PRRSV and SARS-CoV-2

Ting Tong, Wantao Tang, Shaobo Xiao and Jiangong Liang**

1. Materials and methods

1.1 Materials and reagents

Titanium aluminum carbide (Ti_3AlC_2, 98%, 200 mesh) was purchased from Beijing HWRK Chemical Co. Ltd.; hydrofluoric acid (HF, AR, 40%) from Shanghai Macklin Biochemical Co. Ltd.; tetra-n-propylammonium hydroxide (TPAOH, 25%) from Shanghai Shaosu Reagent Co. Ltd.; sodium 3-mercapto-1-propanesulfonate (MPS) from Aladdin Co. Ltd. (Shanghai, China); tetrachloroauric (III) acid hydrate ($HAuCl_4$, AR) from Sinopharm Chemical Reagent Co., Ltd.; human embryonic kidney-239T cells highly expressing ACE2 (HEK-293T-ACE2), human embryonic kidney-239T cells (HEK-293T), and SARS-CoV-2 pseudovirus from Fubio Biomedical Technology Co., Ltd. Other reagents not mentioned are provided by Sigma-Aldrich Co. Ltd. All chemicals and reagents were used directly as received without further purification.

1.2 Characterization

High resolution transmission electron microscope (HRTEM) imaging was performed on a JEM–2100F, STEM/EDS HR-TEM (JEOL, Japan). Powder X-ray diffraction (XRD) was performed on a Bruker diffractometer (Germany, D8 advance) with Cu K_α radiation (λ = 1.5406 Å) at 40 kV and 40 mA. Ultraviolet visible absorption spectra (UV-Vis) were recorded on a UV-2450 spectrophotometer (Shimadzu, Japan). The dynamic light scattering (DLS) and Zeta potential analyses were performed using the Malvern Zeta sizer instrument (Malvern ZEN 3690). The elemental and structural analysis of the samples was performed using an ESCALAB Xi† photoelectron spectrum (XPS) instrument (Thermo Fisher Scientific, USA). The Fourier transform infrared spectra (FT-IR) were recorded by a Nicolet Avatar-330 Fourier transform infrared spectrometer (Thermo Fisher Scientific, USA). The confocal fluorescence images were obtained by an Olympus IX 70 inverted microscope (Olympus, Japan).

1.3 Cells and viruses

MARC-145 cells, HEK-293T cells, and HEK-293T-ACE2 cells (ACE2-expressing 293T cells) were cultured in DMEM/HIGH GLUCOSE (HyClone) containing 10% fetal bovine serum (FBS, PAN) at 37°C with 5% CO_2 in a humidified incubator. PRRSV strain WUH3 (GenBank accession no. HM853673), a

highly pathogenic type 2 (North American) PRRSV, was previously isolated from the brains of pigs suffering from a high fever syndrome in China at the end of 2006 [1]. PRRSV was amplified, and the titer was determined in MARC-145 cells by plaque reduction assay.

1.4 Plaque reduction assay

To determine viral titers, a plaque reduction assay in MARC-145 cells was performed. Briefly, MARC-145 cells were seeded in 6-well plates and cultured until ~100% confluence and then infected with 800 μL of 10-fold serial dilution of virus. After incubation for 1 h, the nonadherent virions were removed, followed by three washes with PBS, and overlaying the cells with 48% low melting agarose (Biofrox, w/v: 1.8%) in DMEM without phenol red (Gibco) supplemented with 3% FBS and 1% kanamycin-streptomycin. Then the 6-well plate was placed at 4 °Cfor 15 min to coagulate the agarose, followed by incubation at 37 °Cfor 2-4 days, and staining the cells with neutral red (3-Amino-7-dimethylamino-2-methylphenazine hydrochloride, Sigma) solution (0.50 mg·mL^{-1}) for 1 h at 37 °C Finally, cells were washed and viral titers were determined by counting the number of plaques and presented in plaque forming units (PFU/mL).

1.5 Western blot assay

After seeding in 6-well plates and culture until ~80% confluence, the MARC-145 cells were treated as described, followed by washing the cells three times with precooled PBS, suspension in lysis buffer (Beyotime, P0013) with protease inhibitors for indicated periods of time, incubation on ice for 10 min and then centrifugation at 12,000 rpm and 4 °Cfor 10 min. After cell lysis, equal amounts of proteins were separated by sodium dodecyl sulfate-polyacrylamide gel electrophoresis (SDS-PAGE, 12% SDS), and the proteins were transferred to polyvinylidene difluoride (PVDF) membranes (0.22 μm, Millipore, Billerica, MA). After blocking for 4 h with 10% (w/v) nonfat dry milk in Tris-buffer containing Tween 20 (TBST), the membranes were incubated with various antibodies, including primary antibodies against PRRSV N protein and PRRSV nsp2 protein for 4 h at room temperature, with β-actin as the internal control (1:1000, A1978, Sigma-Aldrich). After three washes with TBST, the membranes were incubated for 1 h at room temperature with corresponding horseradish-peroxidase (HRP)-conjugated secondary antibodies (Beyotime) against mouse primary antibodies. After another four washes with TBST, the membranes were detected by the ChemiDocTM imager (Bio-Rad) and analyzed using the Image LabTM software (Bio-Rad).

1.6 Indirect immunofluorescence assay

After seeding on circular glass coverslips in 24-well plates, the MARC-145 cells were treated with GFP-PRRSV at a MOI of 1.0. At the indicated time points post infection, cells were fixed with 4% paraformaldehyde for 15 min and then permeabilized with precooled methanol for 10 min. After

blocking with 5% BSA for 1 h, cells were incubated with 4, 6-diamidino-2-phenylindole (DAPI; Beyotime) (5 μg·mL^{-1}) in PBS for 15 min. Finally, the fluorescent images were acquired with an Olympus FV10 laser scanning confocal microscope (Olympus, Tokyo, Japan).

1.7 Quantitative Reverse Transcription-Polymerase Chain Reaction (RT-qPCR)

After seeding in 24-well plates and culture until ~80% confluence, the MARC-145 cells were treated as described**,** followed by extracting the RNA from the samples at indicated time points by using RNA-Solv® Reagent (Omega Bio-tek), and reverse transcription of 1 μg RNA to cDNA with the Transcriptor First Strand cDNA synthesis kit (Roche, Mannheim, Germany) using random primers as instructed by the respective manufacturers. Subsequently, the resulting cDNA was used as the template in a SYBR green qPCR assay (Applied Biosystems), using (primers specific to PRRSV ORF7 gene as well as the PRRSV ORF7 gene) forward and reverse primers of q5'ORF7- F: GCATTTGTATTGTCAGGAGC-3' and q5'ORF7-R: AGCAGTGCAACTCCGGAAG-3', respectively [2]. Each sample was assayed three times. The abundance of individual mRNA transcripts in each sample was assayed three times and normalized to that of GAPDH mRNA (internal control). The forward and reverse primers for the MARC-145 cell reference gene were q5'GAPDH-F: TCATGACCACAGTCCATGCC and q5'GAPDH-R: GGATGACCTTGCCCACAGCC.

1.8 Replication assay

MARC-145 cells were incubated with PRRSV (MOI=1.0) for 6 h, followed by removing the cell-free virus particles and culturing the cells in fresh medium containing Ti_3C_2-Au-MPS (0-200 μg·mL^{-1}). At 7, 8, 9, and 10 hpi, the infected cells were collected and total RNA was isolated using Trizol reagent following the manufacturer's instructions (Invitrogen). After reverse transcription of 1 μg RNA to cDNA with the Transcriptor First Strand cDNA synthesis kit (Roche, Mannheim, Germany) using the primer 5'UF: GACGTATAGGTGTTGGCTC, PRRSV negative-sense RNA was quantified by RT-qPCR using forward and reverse primers of q5'UTR-F: GCATTTGTATTGTCAGGAGC, and q5'UTR-R: AGCAGTGCAACTCCGGAAG. For the MARC-145 cell reference gene, the forward and reverse primers were q5'GAPDH-F: TCATGACCACAGTCCATGCC, and q5'GAPDH-R: GGATGACCTTGCCCACAGCC.

2. Results and discussion

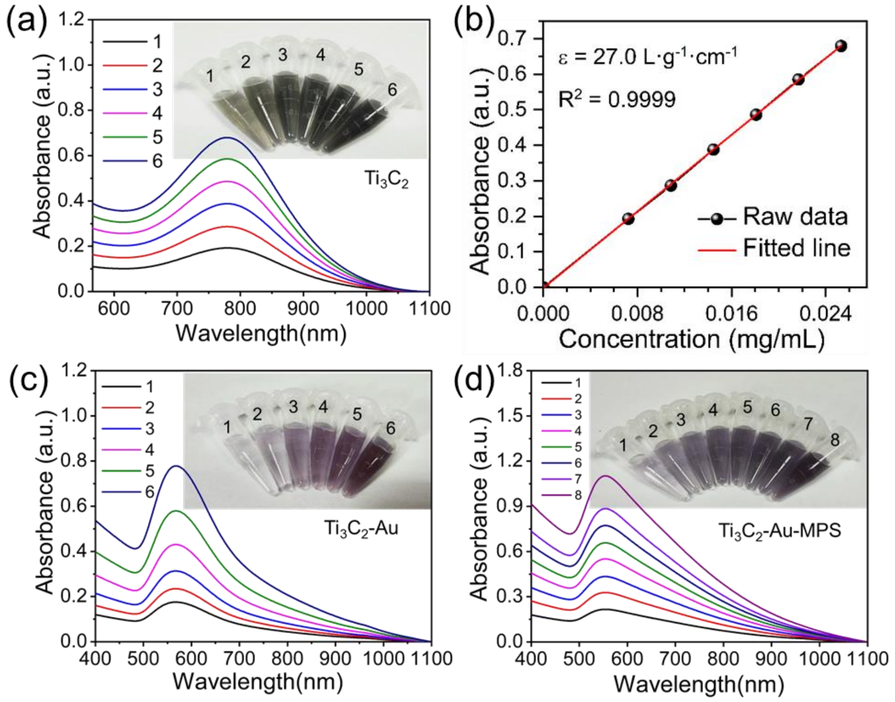

Figure S1. Optical images and UV-Vis spectra. (a) Optical images and UV-Vis spectra of Ti_3C_2 nanosheets. The Ti_3C_2 nanosheets concentrations represented by the numbers 1-6 in Figure (a) are 7.23 μg·mL^{-1}, 10.84 μg·mL^{-1}, 14.46 μg·mL^{-1}, 18.07 μg·mL^{-1}, 21.69 μg·mL^{-1}, and 25.30 μg·mL^{-1}. (b) Extinction coefficient spectrum of Ti_3C_2 nanosheets. (c) Optical images and UV-Vis spectra of Ti_3C_2-Au nanocomposites. The Ti_3C_2-Au nanocomposites concentrations represented by the numbers 1-6 in Figure (c) are 3.60 μg·mL^{-1}, 7.21 μg·mL^{-1}, 14.41 μg·mL^{-1}, 28.83 μg·mL^{-1}, 57.65 μg·mL^{-1}, and 115.31 μg·mL^{-1}. (d) Optical images and UV-Vis spectra of Ti_3C_2-Au-MPS nanocomposites. The Ti_3C_2-Au-MPS nanocomposites concentrations represented by the numbers 1-8 in Figure (d) are18.59 μg·mL^{-1}, 37.18 μg·mL^{-1}, 55.76 μg·mL^{-1}, 74.35 μg·mL^{-1}, 92.94 μg·mL^{-1}, 111.53 μg·mL^{-1}, 130.11 μg·mL^{-1}, and 148.70 μg·mL^{-1}.

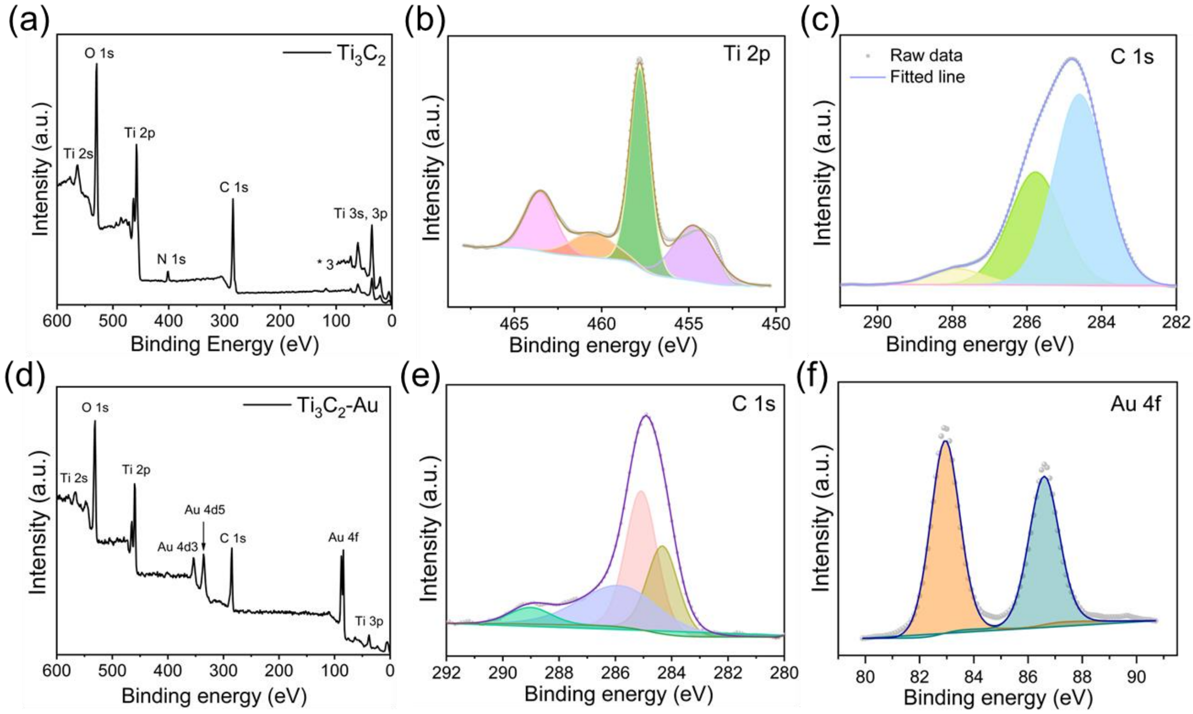

Figure S2. XPS spectra of Ti_3C_2 nanosheets and Ti_3C_2-Au nanocomposites. (a) Full scan XPS spectrum of Ti_3C_2 nanosheets. (b, c) High-resolution XPS spectra of Ti 2p and C 1s of Ti_3C_2 nanosheets. (d) Full scan XPS spectrum of Ti_3C_2-Au nanocomposites. (e, f) High-resolution XPS spectra of C 1s and Au 4f of Ti_3C_2-Au nanocomposites.

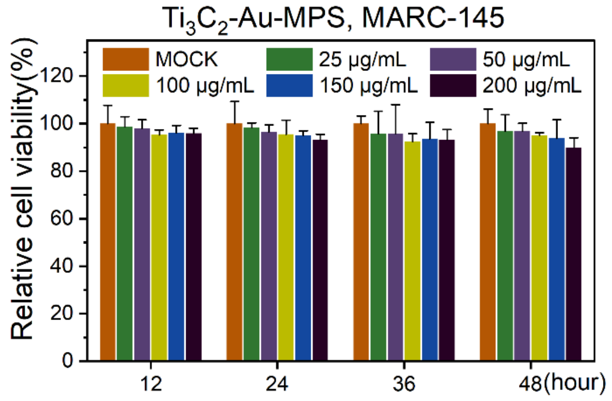

Figure S3. Cytotoxicity of Ti_3C_2-Au-MPS nanocomposites (0-200 $\mu g \cdot mL^{-1}$) on MARC-145 cells for 12, 24, 36 and 48 h by MTT assay. Error bars represent the standard deviation (SD) from three repeated experiments.

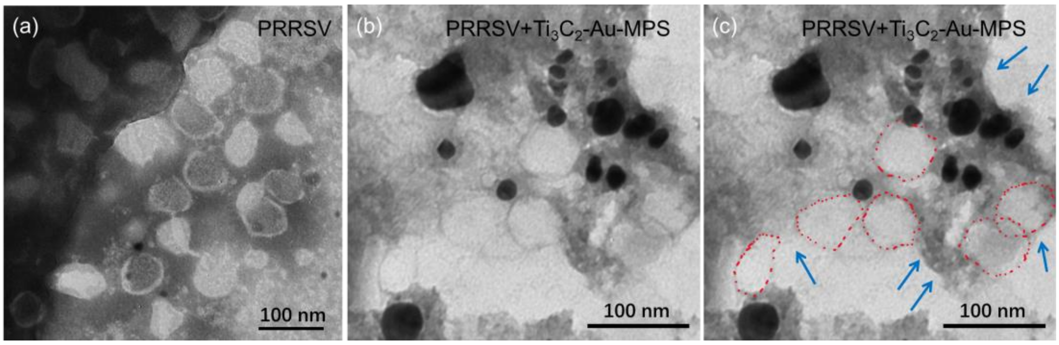

Figure S4. Ti_3C_2-Au-MPS nanocomposites directly interact with PRRS virions and block the viral infectivity. The PRRS virions were negatively stained and characterized by transmission electron microscopy. (a) Individual PRRS virions were seen to present an ellipse morphology with a size of ~ 60 nm and a viral envelope structure with the marked results of **Figure S4b** shown in **Figure S4c**, where PRRS virions are circled by red dotted lines, and Ti_3C_2-Au-MPS nanocomposites are indicated by blue arrows.

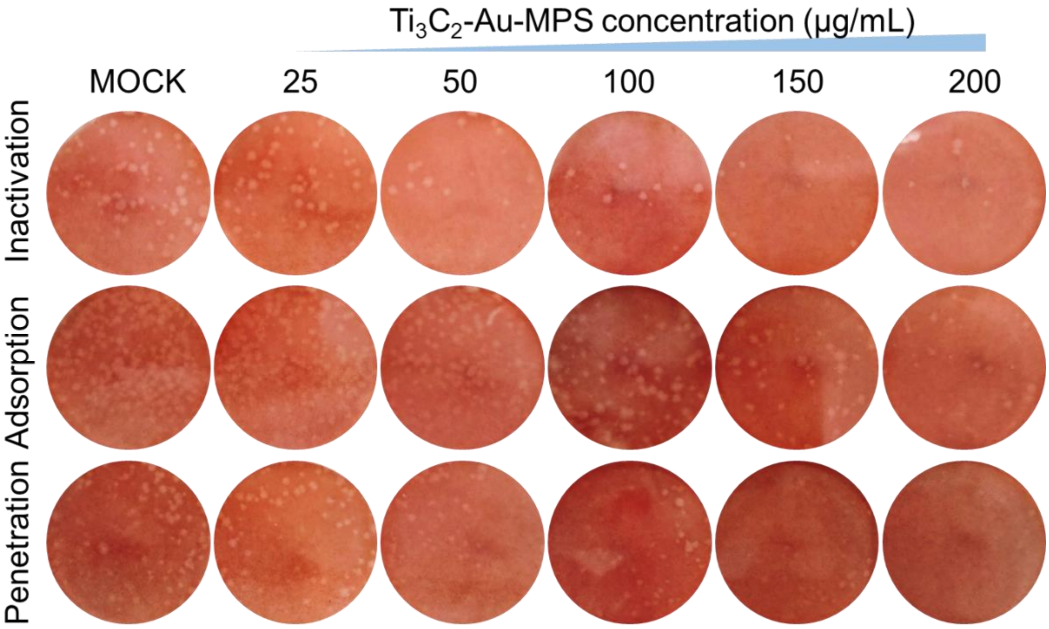

Figure S5. Plaque reduction experiment images for the inhibitory effects of Ti_3C_2-Au-MPS nanocomposites on virus proliferation at different stages (MOI=0.001).

Reference

[1] B. Li, L. Fang, S. Liu, F. Zhao, Y. Jiang, K. He, H. Chen, S. Xiao, *Vet. Microbiol.* **2010**, *146*, 226.

[2] Y. Zhou, T. Tong, X. Jiang, L. Fang, Y. Wu, J. Liang, S. Xiao, *ACS Appl. Bio Mater.* **2020**, *3*, 4809.
